# Cancer-Associated Immune Resistance and Evasion of Immune Surveillance in Colorectal Cancer

**DOI:** 10.1155/2016/6261721

**Published:** 2016-02-23

**Authors:** Pietro Parcesepe, Guido Giordano, Carmelo Laudanna, Antonio Febbraro, Massimo Pancione

**Affiliations:** ^1^Department of Pathology and Diagnostics, University of Verona, 31134 Verona, Italy; ^2^Medical Oncology Unit, Fatebenefratelli Hospital, 82100 Benevento, Italy; ^3^Department of Experimental and Clinical Medicine “Gaetano Salvatore”, University “Magna Grecia”, 88100 Catanzaro, Italy; ^4^Department of Sciences and Technologies, University of Sannio, 82100 Benevento, Italy

## Abstract

Data from molecular profiles of tumors and tumor associated cells provide a model in which cancer cells can acquire the capability of avoiding immune surveillance by expressing an immune-like phenotype. Recent works reveal that expression of immune antigens (PDL1, CD47, CD73, CD14, CD68, MAC387, CD163, DAP12, and CD15) by tumor cells “immune resistance,” combined with prometastatic function of nonmalignant infiltrating cells, may represent a strategy to overcome the rate-limiting steps of metastatic cascade through (a) enhanced interactions with protumorigenic myeloid cells and escape from T-dependent immune response mediated by CD8+ and natural killer (NK) cells; (b) production of immune mediators that establish a local and systemic tumor-supportive environment (premetastatic niche); (c) ability to survive either in the peripheral blood as circulating tumor cells (CTCs) or at the metastatic site forming a cooperative prometastatic loop with foreign “myeloid” cells, macrophages, and neutrophils, respectively. The development of cancer-specific “immune resistance” can be orchestrated either by cooperation with tumor microenvironment or by successive rounds of genetic/epigenetic changes. Recognition of the applicability of this model may provide effective therapeutic avenues for complete elimination of immune-resistant metastatic cells and for enhanced antitumor immunity as part of a combinatorial strategy.

## 1. Introduction

Metastasis remains the most significant cause of cancer-associated morbidity and mortality and specific targeting molecules have had limited success in reversing metastatic progression in the clinical setting [1–3]. Understanding the exact molecular and cellular basis of the events that facilitate cancer metastasis has been difficult so far. Over the past years, a well-accepted theory suggests that genomic alterations of the malignant cells accompanied by the so-called tumor microenvironment “nonmalignant cells” contribute to the metastatic cascade [4, 5]. As such, metastasis is frequently described as the sequential execution of multiple steps. To establish the metastatic tumor, cancer cells have to acquire the traits that enable them to efficiently cooperate with the host stroma and simultaneously avoid antitumor immune response [4–9]. At early stage of carcinogenesis, tumors appear to be vulnerable because mutant and thus potentially immunogenic tumor cells are being exposed to the immune system, which can recognize them and restrict their growth [10, 11]. This is the case of tumor-infiltrating immune cells particularly CD8+ T cells and NK cells which have the potential to restrict the tumor outgrowth or reject metastatic tumor cells [12, 13]. According to this notion, in most primary tumors, a strong Th1/cytotoxic T cells infiltration correlates with a longer patient's survival [12–14]. Unfortunately, tumor develops multiple mechanisms of evading immune responses, by forming a compromised microenvironment that allows the dissemination of malignant cells in a foreign microenvironment through molecular mechanisms still poorly characterized. A variety of stromal cells, particularly M2-phenotype macrophages and myeloid-derived suppressor cells (MDSCs), are recruited to primary tumors; these not only enhance growth of the primary cancer but also facilitate its metastatic dissemination to distant organs [13, 14]. Notably, cooperative “dialogue” between malignant cells and their microenvironment will go on in the systemic circulation and subsequently in the future metastatic site [13–17]. In fact, recent studies have demonstrated that a high systemic inflammatory response, that is, blood neutrophil-lymphocyte ratio (NLR), predicts lower overall survival, higher tumor stage, and a greater incidence of metastasis in multiple tumor types [18, 19]. Therefore, a substantial amount of data suggests a novel dimension of the tumor biology and offers the opportunity to revisit the mechanisms describing evasion of cancer immunosurveillance during the metastatic process. The present review analyses recent studies that elucidate and reinforce the theory by which immune-phenotypic features or “immune resistance” by cancer cells may need to sustain the metastatic cascade and avoid antitumor immune response.

## 2. Tumor Antigens and Antitumor Immune Response by Effectors of Adaptive Immunity

**Figure 1:**
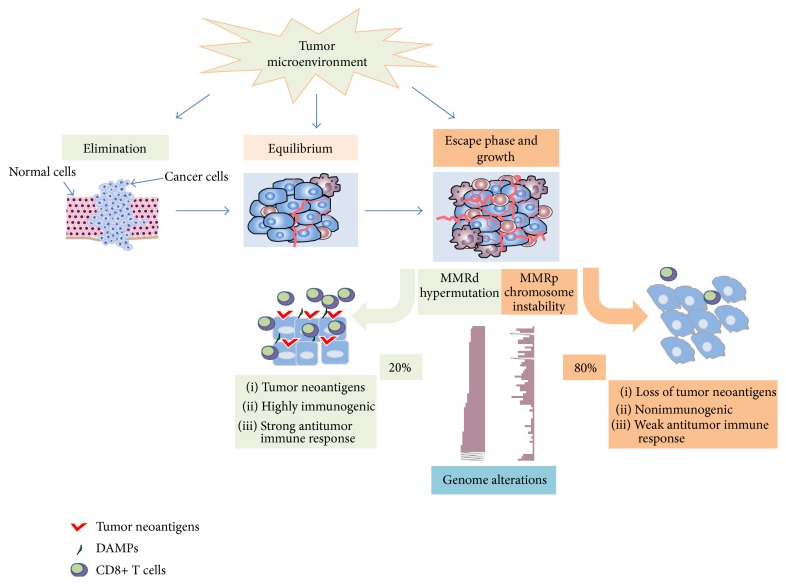
Tumor microenvironment and subversion of immunosurveillance during carcinogenesis. At early stage of primary tumor expansion, the immune system can specifically identify and eliminate tumor cells on the basis of their expression of specific antigens “highly immunogenic tumor.” However, there might not be a complete elimination leading to the survival of some cancer cells that nevertheless remain under immunosurveillance “state of equilibrium.” If the immune response fails to completely eliminate the tumor, cancer cells can avoid or suppress the antitumor immune response leading to a progressively growing tumor. In the case of colorectal cancer (CRC) subtypes, different combinations of genetic and epigenetic changes lead to well distinct microsatellite stable (MMRp) or unstable or MMRd subtypes with different mutational load. Studies have shown that MMRd CRC have a high number of somatic mutations, “hypermutated tumors,” that can give rise to neoepitopes or damage-associated molecular patterns (DAMPs) and that these may serve as neoantigens for activation of tumor-specific CD8 T cell responses. The large majority of CRC are nonhypermutated cases tending to exhibit chromosomal instability associated with loss of tumor antigens, loss of human leukocyte antigen molecules, loss of sensitivity to complement, or T cell lysis, making them a poor target of an immune attack.

A decade of studies has emphasized the nature of cancer as a systemic disease remarking a key role of host microenvironment as a critical hallmark. As a result, a new picture of cancer is emerging in particular due to unexpected cross-talk between malignant cells and the immune system [3–5]. Recent data have expanded the mechanisms of cancer-immune system interactions revealing that every known innate and adaptive immune effector component participates in tumor recognition and control [9, 10]. It is now recognized that in different individuals and with different cancers, at early stage of tumorigenesis, the few cancer cells are detected by NK cells through their encounter with specific ligands on tumor cells [5]. In turn, activation of macrophages and dendritic cells and particularly T and B cells expands production of additional cytokines and further promotes activation of tumor-specific T cells “CD8+ cytotoxic T cells” leading to the generation of immune memory to specific tumor components [14–16]. However, in cases where the immune system is not able to eliminate the cancer, a state of equilibrium develops or eventually cancer cells can resist, avoid, or suppress the antitumor immune response, leading to the immune escape and a fully developed tumor (Figure 1) [9–15]. For example, investigations into the nature of cancer as a genetic disease have suggested two paradigmatic subtypes of colorectal cancer (CRC): chromosomal instability (CIN) and microsatellite instability (MSI), in which the expression of immune-checkpoint proteins can be differentially dysregulated to unleash the potential of the antitumor immune response [11]. In particular, tumors with mismatch-repair deficiency (dMMR) (10–20%) of advanced colorectal cancer tend to have 10 to 100 times more somatic mutations and higher amount of lymphocyte infiltrates than mismatch-repair-proficient colorectal cancers (pMMR), a finding consistent with a stronger antitumor immune response (Figure 1) [11, 20]. According to this notion, recent studies suggest that certain cancer subtypes dMMR CRC with high numbers of somatic mutations are more responsive to PD-1 blockade, a well-known immune-checkpoint inhibitor [20]. In particular, CD8-positive lymphoid infiltrate and membranous PDL1 expression on either tumor cells or tumor-infiltrating lymphocytes at the invasive fronts of the tumor are associated with an improved response to anti-PD-1 therapy in patients with mismatch-repair-deficient cancer [11, 20]. In addition, cancer subtypes with stronger antitumor immune responses (immunogenic) are characterized by surface-exposed calreticulin or heat shock protein 90 (HSP90), which serve as a powerful mobilizing signal to the immune system in the context of damage-associated molecular patterns (DAMPs) [17]. As danger signals, DAMPs accompanied by subversion MHC Class I and II antigens on the plasma membrane of cancer cells appear to be characteristic of stressed or injured cells and can act as adjuvant signals to enhance antitumor immunity mediated by the innate immune system [17]. As described in this review, unfortunately, the large majority of human tumors can suppress the immune system to enhance their survival, rendering them invisible to cytotoxic T lymphocytes through a variety of mechanisms. Furthermore, in most cases, tumor-infiltrating immune cells differentiate into cells that promote each step of the tumor progression supporting ability of cancer cells to invade and survive in foreign organs. In addition, the intricate network of malignant and immune components represents a prominent obstacle to the effects of therapeutic agents.

## 3. Tumor-Infiltrating Immune Cells and Immune Escape Mechanisms

**Figure 2:**
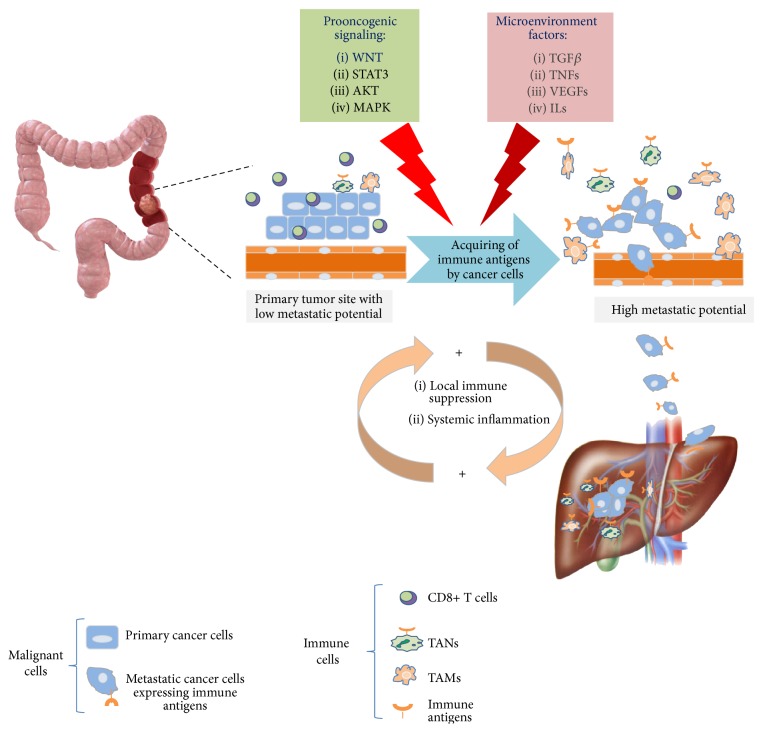
Immune antigens expression by cancer cells contributes to the metastatic cascade. Most malignant solid tumors metastasize from the primary organ to another, such as the liver. At the primary site, potentially immunogenic tumor cells are being exposed to the immune system. High density of TILs and tumoricidal immune response mediated by killer cells and CD8+ T cells restrict the tumor outgrowth. Genetic damage and deregulated signaling pathways accompanied by the production of various cytokines and chemokines in the tumor microenvironment can induce expression of immune antigens on the cancer cell surface. By these changes, the tumors promote recruitment and expansion of prometastatic myeloid cells, TAMs, or TANs, respectively. As such, immune-like phenotype enables cancer cells to increase metastatic potential and avoid immune surveillance at different levels during the metastatic cascade. The circulating tumor cells expressing an immune-like phenotype, that is, CD47, are then arrested in microvessels in the metastatic site where they need to survive. At the metastatic site, the arrested tumor cells escape from the resident myeloid cells and survive at the metastatic niche and proliferate to form the deadly metastatic tumor. Recent studies suggest that MAMs sustain the survival and persistent growth of emigrated cancer cells. TANs also promote the entrapment of circulating cancer cells by producing neutrophil extracellular traps (NETs). TAMs: tumor associated macrophages; MAMs: metastasis-associated macrophages; TANs: tumor associated neutrophils.

Tumors develop numerous strategies to avoid detection and eradication by the immune system. Among these, one of the best known includes the recruitment, expansion, and function of tumor-infiltrating leukocytes, such as immunoregulatory myeloid cells, that is, regulatory T cells (Treg cells) and T helper 17 cells (TH17 cells) [13–17]. In a variety of cancers, the transition from precancerous to invasive stage parallels a shift from Th1 to Th2 immune microenvironment. However, what makes the same T cell subset (e.g., CD8+ T cells) antitumorigenic in one cancer and protumorigenic in another remains largely unknown [13–17]. An increased number of T cells, including specifically activated CD8+ cytotoxic T cells (CTLs) and natural killer T (NKT) cells, generally correlate with better survival in some cancers as well as in patients with mismatch-repair-deficient colorectal cancer [13–17]. Accordingly, depletion of CD8+ T cells and NK cells consequently increases cancer metastasis without affecting primary tumor growth suggesting that cytotoxic lymphocytes have also metastasis-inhibiting effects [13]. The inflammatory contexts can promote tumor growth through the production of cytokines such as IL-6, IL-1, or TNF-a and angiogenic molecules such as VEGFA, placental growth factor (PlGF), or transforming growth factor-b (TGF-b) (Figure 2) [13–19, 22]. In addition, the so-called adenosinergic signaling cascade “adenosine production” mediated by CD39 and CD73 further promotes immune suppression as well as prostaglandin E2 and suppressor myeloid or T cells attracting chemokines, including CXCL1, CXCL5, CCL2, and CCL12 [13, 14, 17]. This inflammatory milieu contributes to recruiting myeloid cells particularly the macrophages also named tumor associated macrophages (TAMs). They are recognized as the most common type of immune infiltrating cells protumorigenic in the tumor microenvironment (TME) in the primary tumors [15, 16]. Accumulating data suggest that TME polarizes recruited macrophages from a potentially tumor-reactive state (M1) to a tumor-promoting state (M2) phenotype [16]. TAMs can also exert prometastatic functions, by suppressing the cytotoxic activity of CD8+ T cells in primary tumors [13]. A type of immature myeloid cell that expresses CD11b and GR1 is also found in the TME. These cells can suppress the proliferation and the cytokine production of T cells* in vitro* and are thus referred to as myeloid-derived suppressor cells (MDSCs). Similar to macrophages, tumor associated neutrophils (TANs) with “N2 phenotype” can reduce CD8+ T cell activity and increase primary tumor growth (Figure 2). Interestingly, the prognostic role of elevated blood neutrophils and elevated blood neutrophil/lymphocyte ratio has been associated with poor clinical outcome [18, 19]. Instead, in the case of tumor-infiltrating neutrophils, recent studies suggest that high levels of myeloid cells CD33+HLA-DR−CD16+ are associated with improved colorectal cancer (CRC) prognosis [23–26]. Consistently, a high density of MPO+ or CD15+ infiltrating cells consistent with the phenotype of the granulocytic lineage cells has been shown to be of benefit for CRC patient [26]. The role of neutrophil granulocytes (NGs) in the context of cancer-related inflammation remains matter of debate, even if their clinical relevance has recently begun to emerge.

## 4. Generation of a Tumor Microenvironment Permissive for Metastatic Cascade

**Figure 3:**
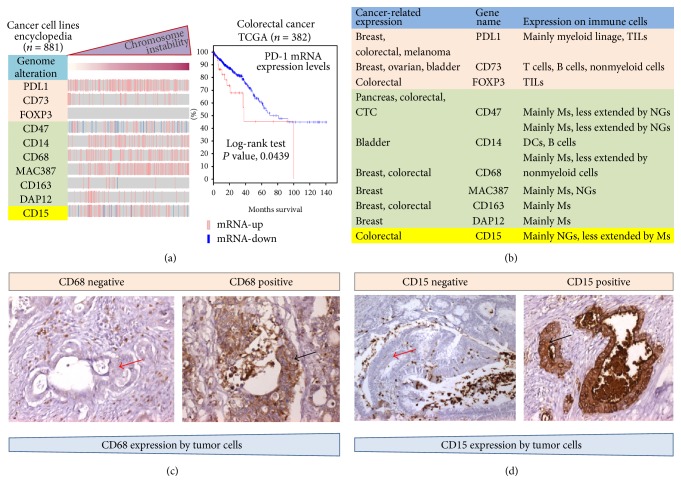
Subsets of immune-phenotypic genes correlate with genome instability in a variety of tumors. (a) Genome instability represents one of the most important hallmarks of genetic diversity in tumors. We utilized a panel of 881 human tumor cell lines derived from different tissues of origin that has been extensively characterized for gene expression and copy-number variations and commonly used for genetic analysis and screening of potential chemotherapeutic agents [21]. mRNA expression of the immune antigens subdivided into low and high is shown. Kaplan-Meier curve shows that, compared with tumors expressing low* PD-1* mRNA levels (blue line, 348/382, 91%), tumors with high* PD-1* mRNA expression (red line, 34/382, 9%) are significantly associated with poor overall survival by interrogating the public colorectal cancer TCGA data set (*N* = 382). (b) Subsets of immune antigens and their prevalence in cancer cells in solid tumors are reported. (c, d) Immunohistochemical staining of colon cancer with neutrophils and macrophage antigens, CD15 and CD68, respectively. Black arrows indicate the immunostaining on the surface of malignant colonic cells. Red arrows indicate absence of immune antigen positivity, magnification 10x. TILs: tumor-infiltrating lymphocytes; Ms: macrophages; NGs: neutrophil granulocytes; CTCs: circulating tumor cells; DCs: dendritic cells.

Once a cancer is established, the “dialogue” between malignant cells and their microenvironment progressively will go on up to reaching a cooperative interaction. As such, tumor-stromal interactions could help to avoid antitumor immune response and generate the so-called “tumor microenvironment permissive for metastasis” [13]. It is well known that the tumor activates certain immune-checkpoint pathways as a major mechanism of immune resistance, particularly against T cells that are specific for tumor antigens. By upregulating cytotoxic T-lymphocyte-associated antigen 4 (CTLA4) and programmed cell death protein 1 (PD1), tumor cells block antitumor immune responses. Our unpublished observations indicate that tumors with high* PD-1* mRNA expression are significantly associated with poor overall survival in colorectal cancer (Figure 3(a)). Therefore, actually, immune-checkpoint inhibitors against CTLA4 and PD1 are promising new approaches for tackling solid tumors [27–30]. In spite of this, the precise molecular mechanism by which cancer cells acquire the ability to suppress immune responses, evade immune system eradication, and contribute to the metastatic cascade remains still unknown. Numerous studies imply that genetic mutations of metastatic cancers do not significantly differ from their primary tumor [31]. Genomic profiling, second-generation sequencing, and proteomics have dramatically accelerated the effort to comprehensively characterize metastatic tumor cells and to understand their natural history of evolution from primary tumors. New observations have contributed to solidifying a mechanistic concept that a variety of immune-phenotypic antigens can be expressed on the tumor cell surface in many solid tumors (Figures 3(a)–3(d)) [21, 32–39]. Acquisition of such a characteristic by cancer cells is generally correlated to early distant recurrence, local recurrence, and reduced survival time in several tumors, that is, breast, colorectal, and pancreas [21, 32–39]. Thus, cancer-immune-like phenotype in the TME might be relevant to enhance the cooperation with prometastatic immune cells either by contact or by the production of paracrine/autocrine immune-suppressive mediators (Figure 2). This aberrant phenotype may simultaneously inhibit antitumor immune responses and promote metastatic disease at different levels.

## 5. Tumor Immune Resistance: A Model of Metastatic Immune Escape

Over 100 years ago, the English surgeon Stephen Paget described the “seed and soil” theory of metastasis likening tumor cells to “seeds” that are systemically distributed and able to only inhabit particular microenvironment, the “soils.” In the last years, we have begun to reveal fundamental concepts that sustain Paget's hypothesis, pointing to the existence of strategies whereby cancer cells evade immune effectors.

The discovery of effective metastasis-targeting agents (immune-checkpoint inhibitors) that specifically interrupt the communication between cancer cells and their microenvironment has the potential to produce durable clinical responses [30]. Then, how does cancer cell disseminate in several foreign microenvironments? Cancer cells employ several strategies to escape their site of origin and survive in potentially hostile microenvironments, many of which remain still poorly understood. Recent studies revealed that the pattern of the tumor microenvironment remains a major prognostic factor even in the metastatic lesions. Tumor-infiltrating immune cells differentiate into cells that promote each step of the metastatic cascade. In particular, myeloid cells such as tumor associated macrophages (TAMs) and tumor associated neutrophils (TANs) contribute to establishing and maintaining metastatic foci. Several environmental factors, including interleukin-4 (IL-4), tumor-derived transforming growth factor-*β* (TGF*β*), and macrophage migration inhibitory factor (MIF), create a permissive tumor microenvironment for metastasis (TMEM) where the cancer cells intravasate [21, 30, 36].

Further lines of evidence support the role of genome instability, which generates the genetic diversity and consequently can expedite the acquisition of a repertoire of macrophage, neutrophil, or T lymphocyte antigens by cancer cells (Figures 1 and 3(a)) [21, 30, 36]. Multiple general mechanisms are emerging. (a) constitutive oncogenic signaling can upregulate expression of immune genes on tumor cell surface, independently of TME. For example, activation of the AKT and signal transducer and activator of transcription 3 (STAT3) pathways has been reported to drive PDL1 expression, which inhibits local antitumor T cell-mediated responses by direct contact with PD1. This latter is expressed on a large proportion of tumor-infiltrating lymphocytes (TILs); high expression levels of PDL1 on either tumor cells or TILs associate with poor prognosis relative to PDL1 negative tumors [30]. In colorectal carcinoma, membranous PDL1 expression correlates with CD8-positive lymphoid infiltrate located at the invasive fronts of the tumor only in patients with mismatch-repair-deficient cancer. However, expression of CD8 and PD-L1 was not significantly associated with progression-free survival or overall survival [20], suggesting that further studies are needed to explain such results. (b) Prooncogenic signaling can also alter the balance between ATP/ADP, AMP, and adenosine, crucial for tumor progression. The best-understood mechanism includes overexpression of CD73 on cancer cells that plays a crucial role in the adenosinergic signaling by producing adenosine, one of the most important immunosuppressive regulatory molecules in the TME [39]. (c) Alternatively, upregulation of immune antigens is induced on tumor cells in response to cytokines such as interferons (IFNs) following an adaptive response to endogenous antitumor immunity (Figure 2) [39]. At the primary tumor site, these mechanisms allow cancer cells to resist immune elimination and might represent an alternative to the conventional drug resistance mechanisms that involve the mutation of drug targets. However, recent studies also suggest that ectopic immune antigens expressed on the cell surface of malignant cells may help to overcome the rate-limiting steps of metastasis including the need to survive in the blood circulation either as “circulating tumor cells” (CTCs) or by facilitating “retention” at distant metastatic sites [37]. The process of extravasation, retention, and persistent growth of emigrated cancer cells may be supported by direct interactions with the so-called metastasis-associated macrophages (MAMs) or resident neutrophils in foreign organs (Figure 2). Indeed, transcriptomic analyses from CTCs have revealed a marked overexpression of CD47, which is recognized to be a specific macrophage antigen [37]. Thus, it has been proposed that the “do not eat me” signal on cancer cells communicates to the signal regulatory protein-*α* on macrophages and cytotoxic T lymphocytes and prevents their phagocytosis. In addition, expression of nonimmunogenic phenotype by CTC could help to overcome the hostile blood stream environment for tumor cells during the metastatic dissemination [37]. Macrophage traits in tumor cells appear to be the most common and include the expression of the following antigens: CD14, CD68, MAC387, CD163, and DAP12 (Figures 3(b) and 3(c)). However, immune-phenotypic features of the tumor cells are not restricted to the macrophage lineage. To shed light on these aspects, we have recently investigated the clinical significance of a series of immune-phenotypic markers expressed on malignant cells from metastatic CRC patients receiving first-line therapy with targeting agents cetuximab and bevacizumab, respectively [40]. Strikingly, not only did this approach confirm previous links with macrophages antigens, but it also revealed an unexpected connection with NG antigen CD15 protein encoded by* FUT4* gene (Figure 3(d)) [40, 41]. In fact, increased expression of CD15 on tumor cells reflected low levels of intratumoral CD8+ TILs and high systemic inflammation and predicted poorer outcomes in terms of progression-free survival. According to this finding,* CD15* was consistently higher in pMMR than in dMMR colorectal cancer. The subsequent bioinformatic prediction was intriguing, because it revealed that CD15 overexpression by tumor cells is subjected to immune resistance as driven by constitutive oncogenic RAF-MEK-ERK kinase signaling pathways through involvement of prooncogenic receptors* ERBB3* or HER3 and* FGFR4* activation [40]. In line with this, ERBB3 acts as a major cause of treatment failure in cancer therapy, mainly through activation of the PI-3 K/AKT, MEK/MAPK, and Jak/Stat signaling pathways [42]. The molecular mechanisms underlying expression of immune antigens in cancers remain still poorly understood. We cannot exclude the contribution of genetic exchange mediated-cell fusion or genetic exchange between the cells by exosome-mediated transfer. The neutralization of signals through these ectopic antigens might enhance T cell responses to eliminate tumor cells. Another possible therapeutic strategy is withdrawal of microenvironmental support for metastatic cancer cells by targeting prometastatic immune cells, in particular macrophages. Thus, a more detailed understanding of the effector mechanisms and the essential cellular interactions between the various cell types in the TME is needed to allow for more precise therapeutic intervention.

## 6. Concluding Remarks

Over the past decade, the scenario of metastatic process is changing. Studies on the molecular profiles of tumors and tumor associated cells imply that metastatic potential becomes an active process at early stage in cancer progression. Current results indicate that tumors predominantly recruit specific types of suppressor cells to evade antimetastatic immune responses. For metastasis to occur from solid malignancies, tumor cells need to undergo a series of successive rounds of genetic/epigenetic mutation and selection. As such, a key step in the initiation of metastatic cascade may be the selection of nonimmunogenic tumor cell variants in the primary tumor. The data discussed in this review seek to consolidate a new scenario of the tumor biology, in which aberrant immune-phenotypic features expressed by cancer cells contribute to the metastatic cascade evading antitumor immune response. This putative “immune-like phenotype” might be imposed by persistent genetic/epigenetic alterations of the cancer cells and orchestrated by prometastatic function of nonmalignant infiltrating cells. As such cancer-specific immunosubversion protects emigrated cancer cells from surveillance by killer cells and permits the recruitment of immunosuppressive cells in foreign microenvironments through “do not eat me signals” or the so-called homotypic direct or indirect interactions. Detailed knowledge underlying these processes might have important implications to shed light on the impact of tumor heterogeneity on cancer outcomes. They might provide novel platforms to the design of novel immunotherapies aimed at properly activating T cells or modulating inflammatory and immunosuppressive elements. Recognition of the widespread applicability of these concepts could be of help either in the definition of surrogate immune-checkpoint biomarkers that dominate in a particular tumor or as guide for the choice of personalized immune inhibitor molecules.
